# Supplementary material for: Exploring the Optoelectronic Properties of D-A and A-D-A 2,2′-bi[3,2-b]thienothiophene Derivatives
Source: Molecules. 2022 Dec 2;27(23):8463. doi: 10.3390/molecules27238463 (PMC9739722; doi:10.3390/molecules27238463)
Supplement: Supplementary file 1 [file molecules-27-08463-s001.zip › molecules-2047120-supplementary.pdf]

Exploring the optoelectronic properties of D-A and A-D-A 2,2'-bi[3,2-*b*]thienothiophene derivatives

Levi Gabrian, Gavril-Ionel Giurgi, Ioan Stroia, Elena Bogdan, Andreea Petronela Crişan, Niculina Daniela Hădade, Ion Grosu and Anamaria Terec

Contents

^{1}H-NMR, ^{13}C-NMR, HRMS spectra of compounds 4-11 1
Computational details 13
Table S1. HOMO-LUMO energy levels for the [3,2-*b*]-TT core and electron-withdrawing groups 13
Table S2. DFT coordinates for compounds 7-11 13
Figure S24 20

^{1}H-NMR, ^{13}C-NMR, HRMS spectra of compounds 4-11

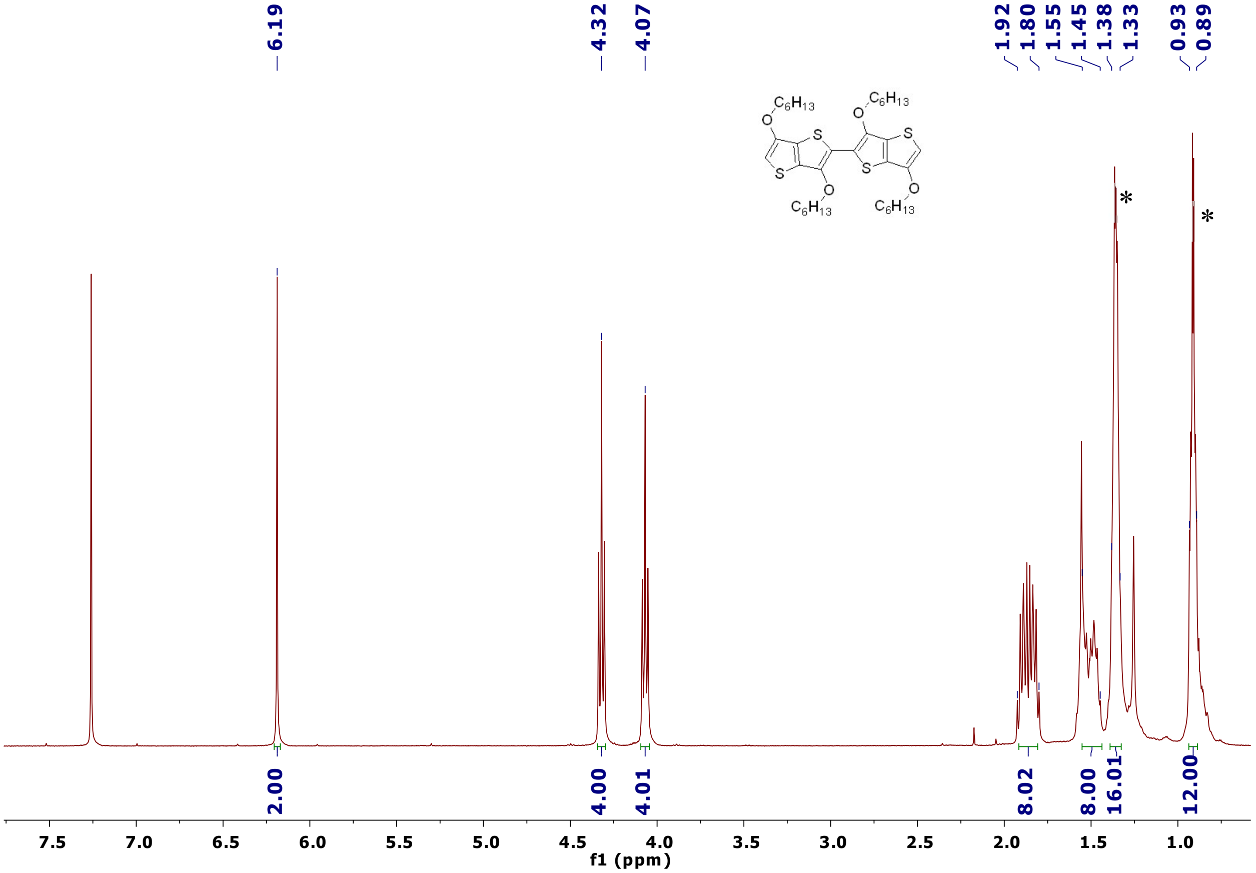

Figure S1 – ^{1}H-NMR spectrum fragment (400 MHz, $CDCl_3$) of **4** (*solvent residue)

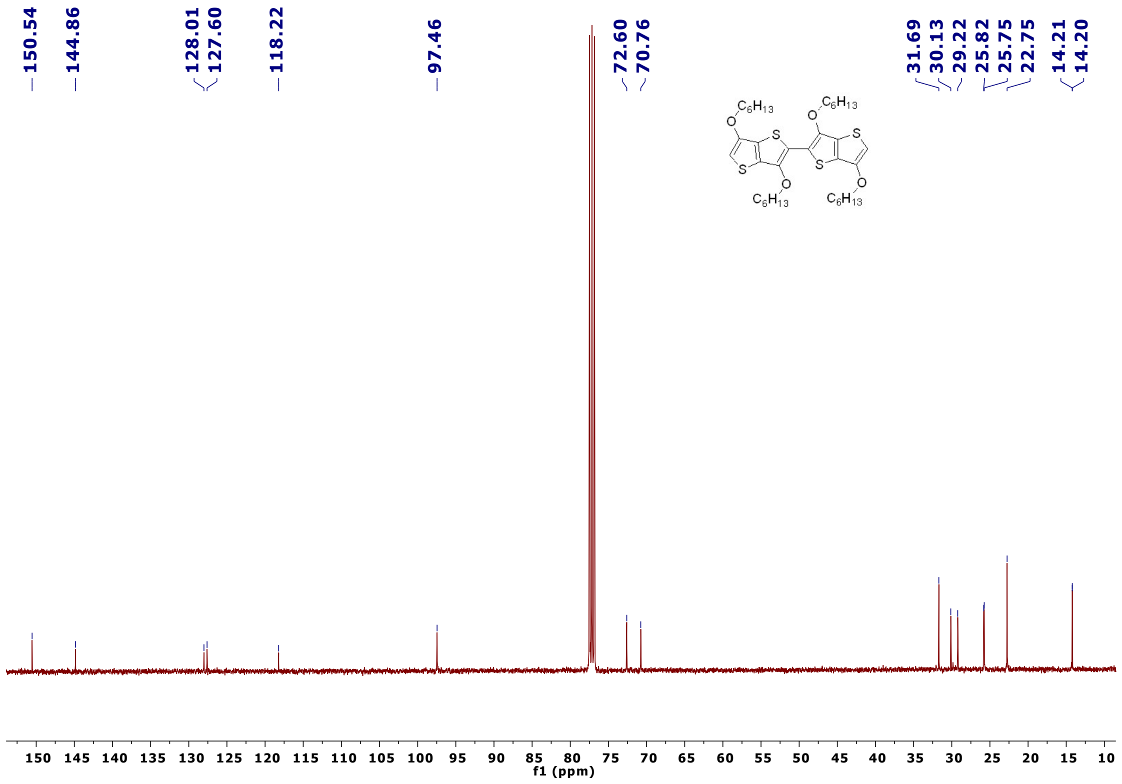

Figure S2 – ^{13}C-NMR spectrum fragment (100 MHz, $CDCl_3$) of **4**

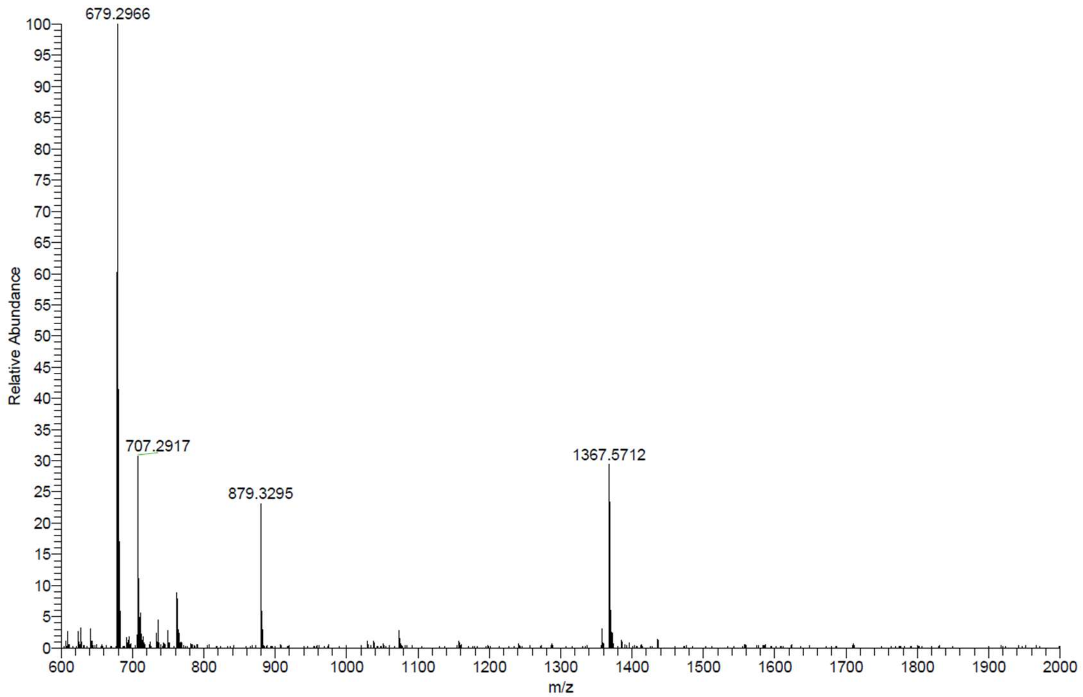

Figure S3 – HRMS spectrum fragment (ESI+) of **4**

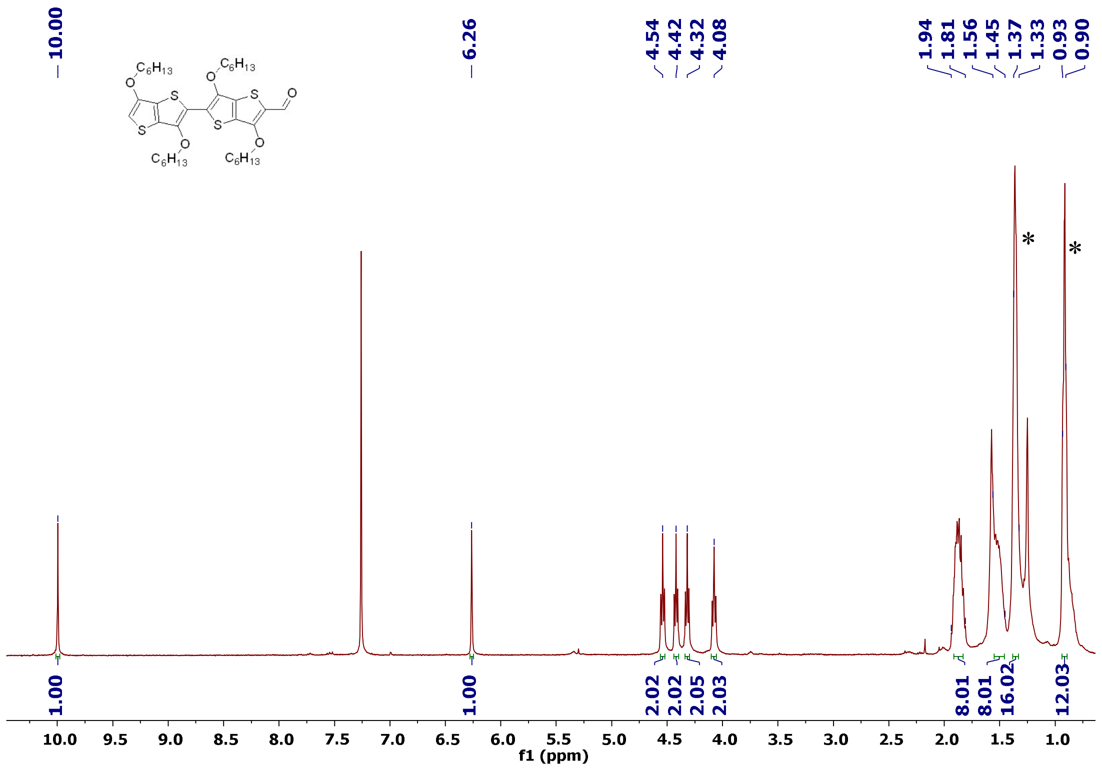

Figure S4 – ^{1}H-NMR spectrum fragment (400 MHz, $CDCl_3$) of **5** (*solvent residue)

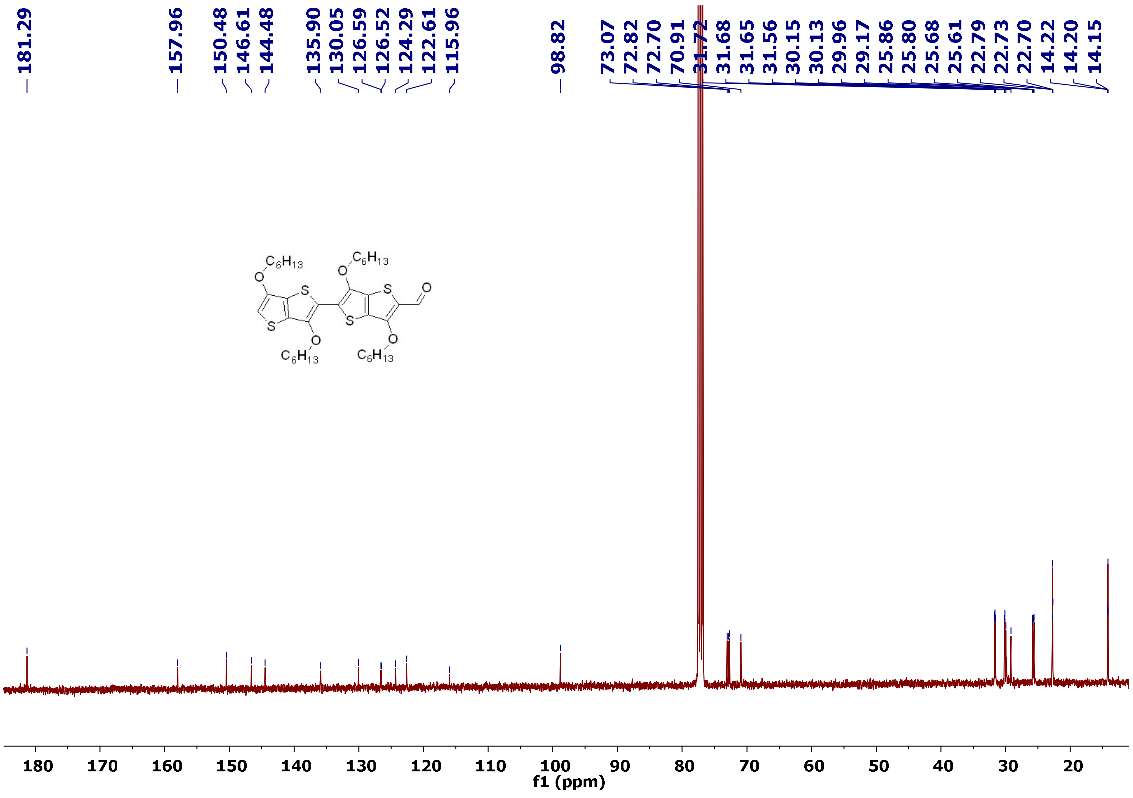

Figure S5 – ^{13}C-NMR spectrum fragment (100 MHz, $CDCl_3$) of **5**

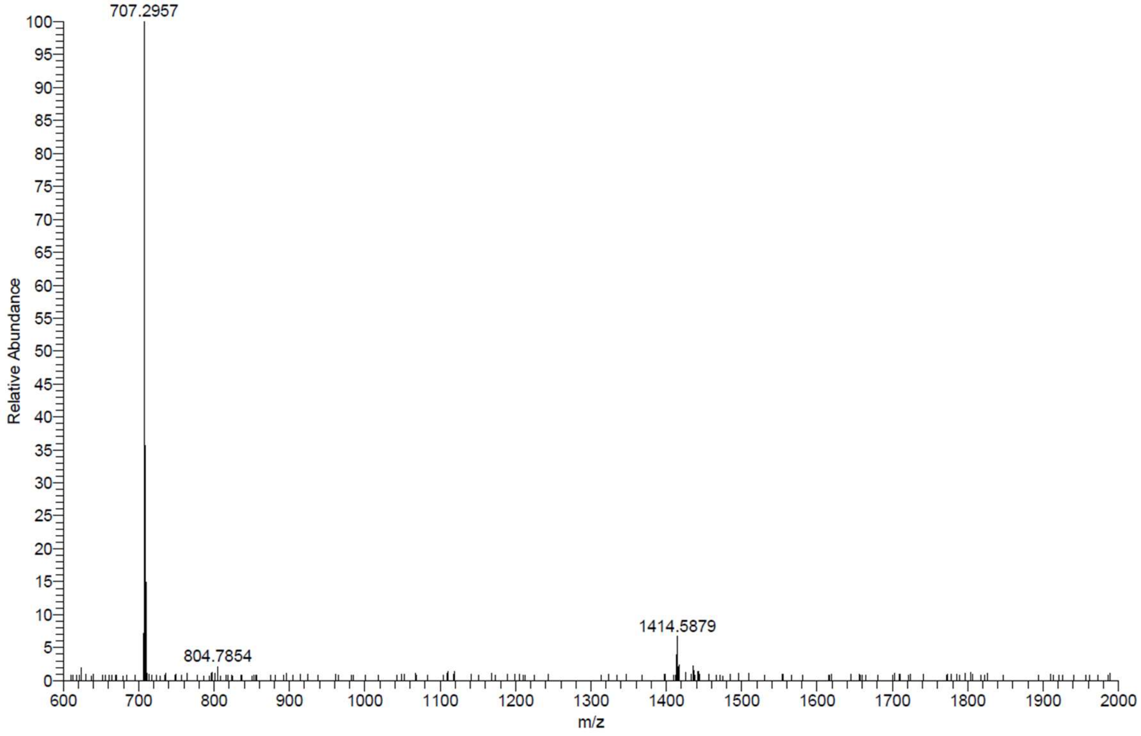

Figure S6 – HRMS spectrum fragment (ESI+) of **5**

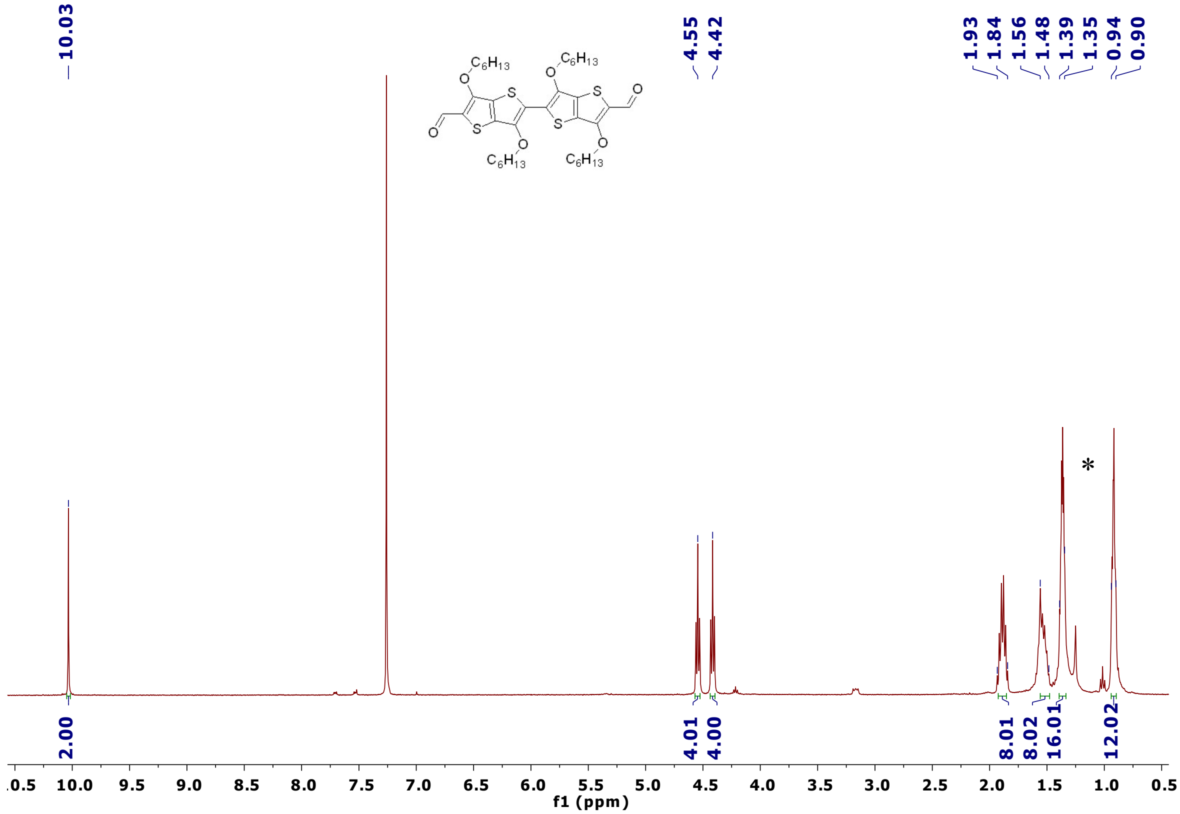

Figure S7 – ^{1}H-NMR spectrum fragment (400 MHz, $CDCl_3$) of **6** (*solvent residue)

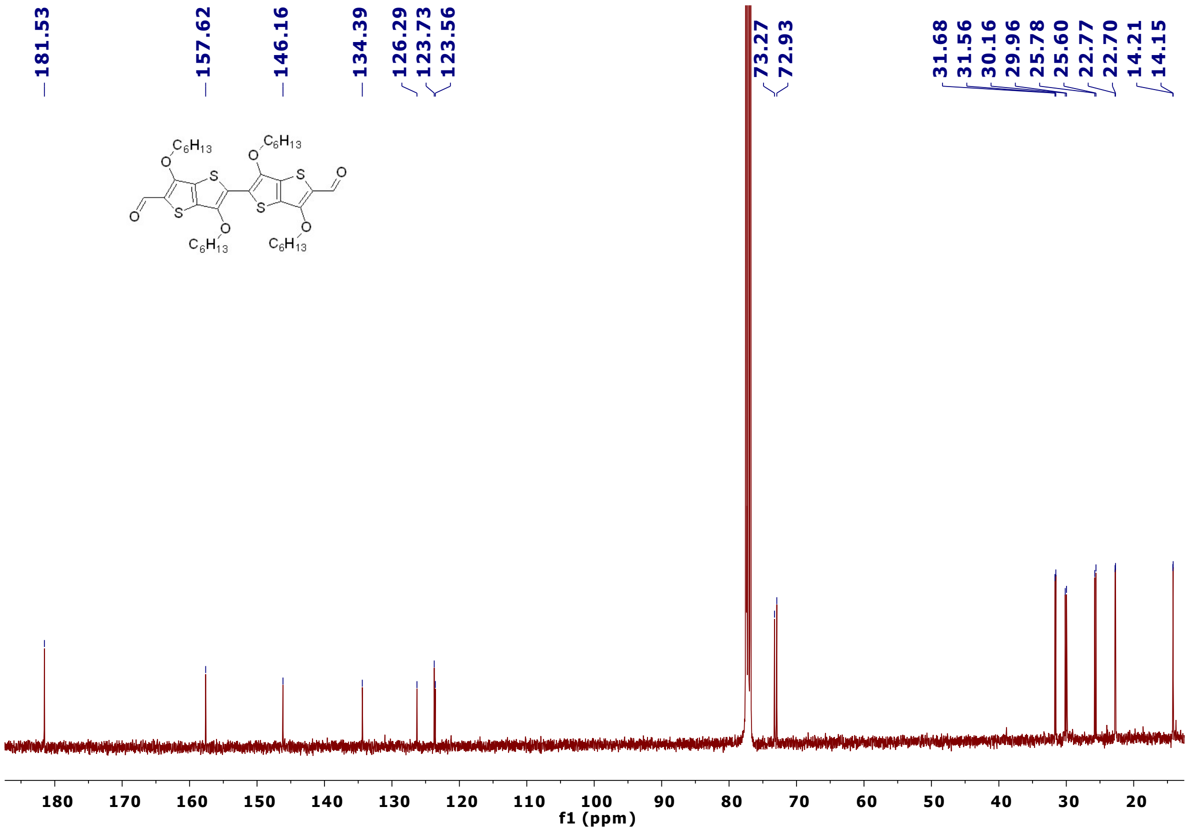

Figure S8 – ^{13}C-NMR spectrum fragment (100 MHz, $CDCl_3$) of **6**

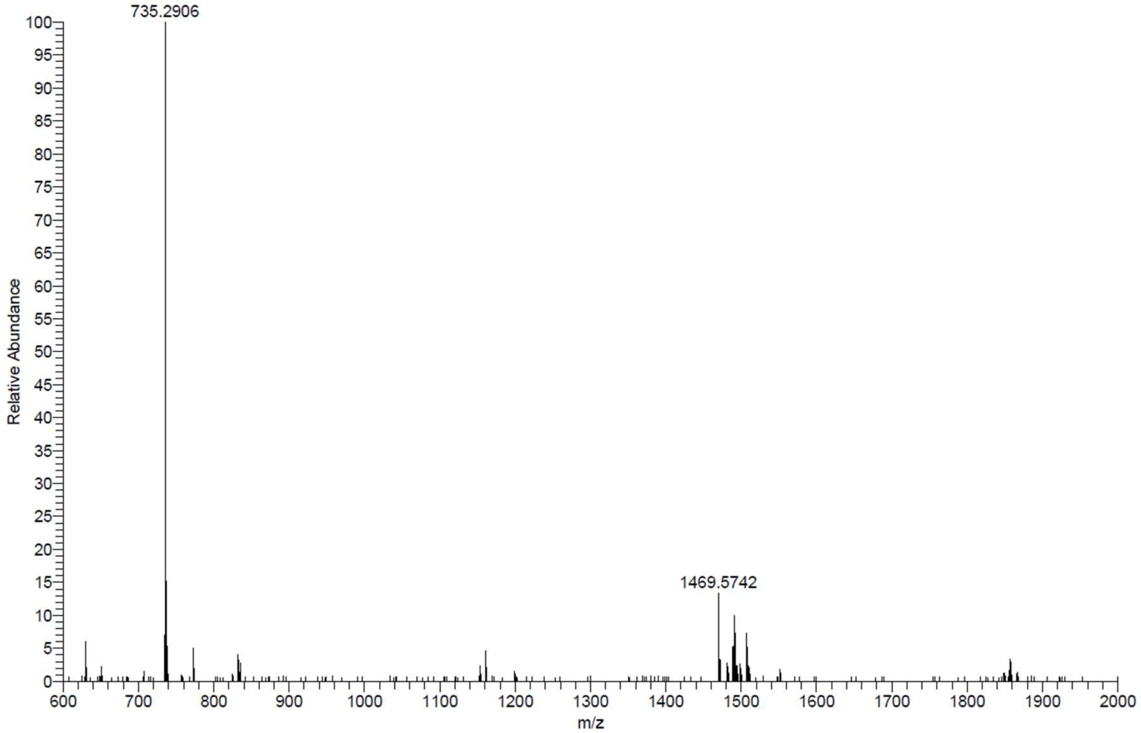

Figure S9 – HRMS spectrum fragment (ESI+) of **6**

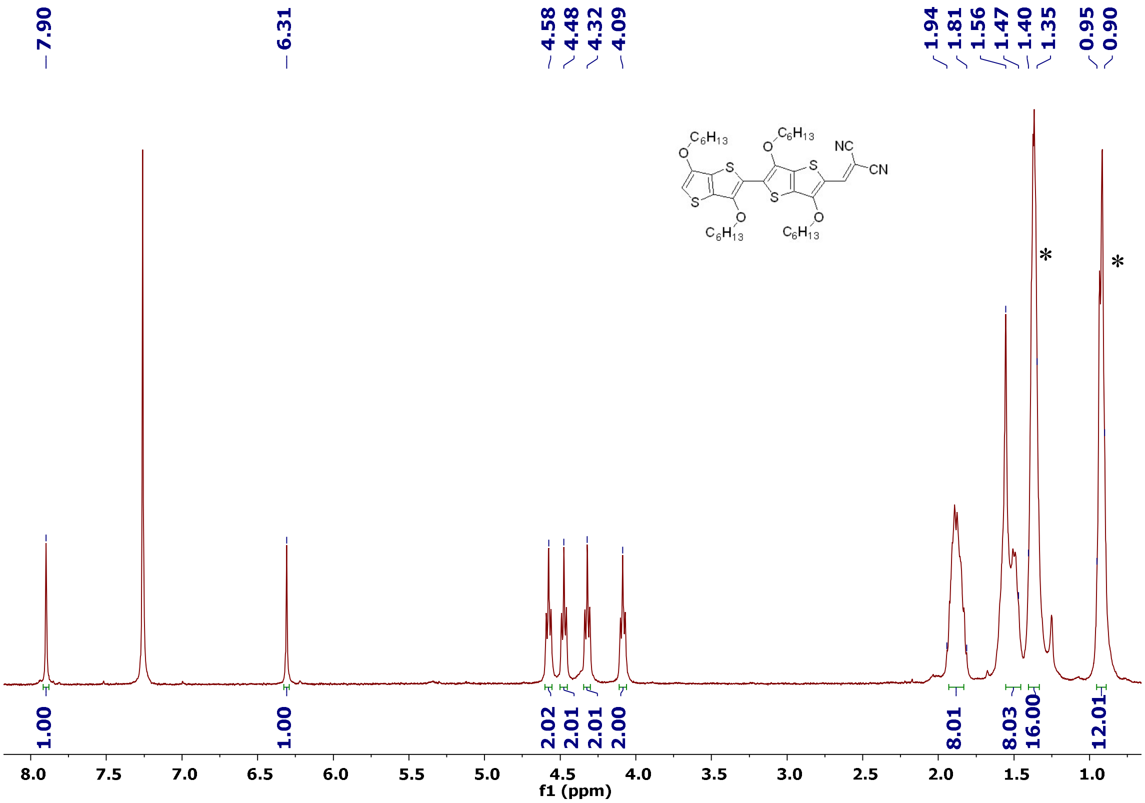

Figure S10 – ^{1}H-NMR spectrum fragment (400 MHz, $CDCl_3$) of **7** (*solvent residue)

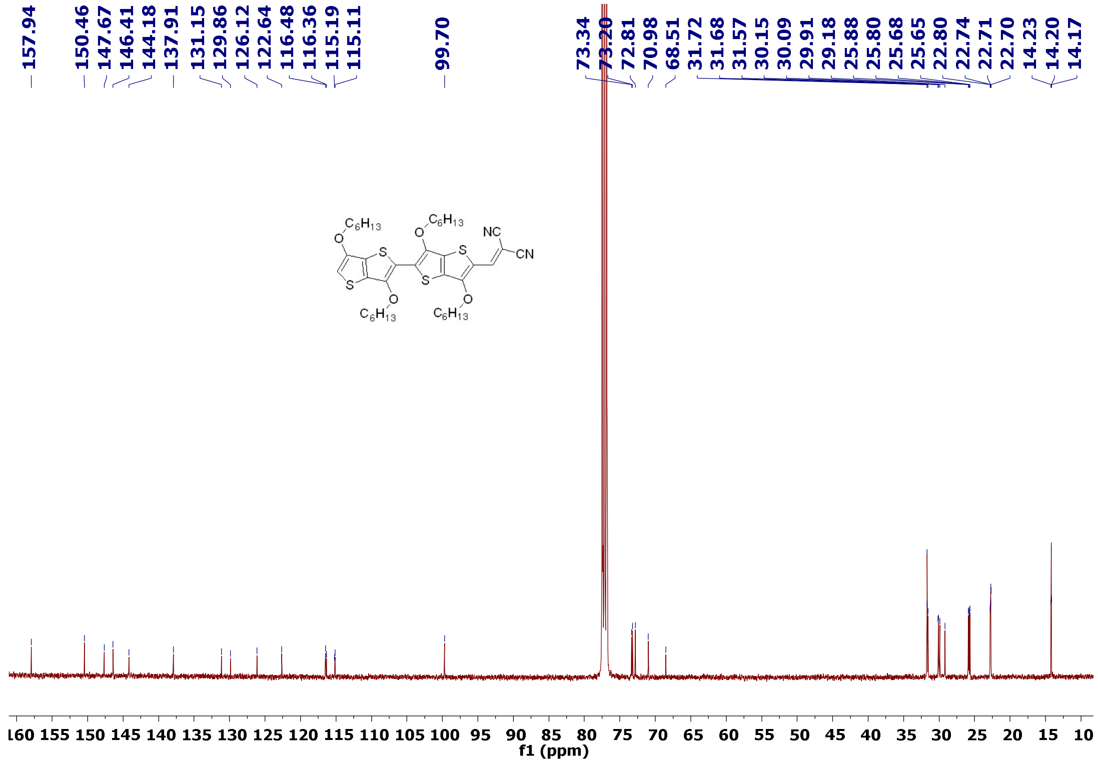

Figure S11 – ^{13}C-NMR spectrum fragment (100 MHz, $CDCl_3$) of **7**

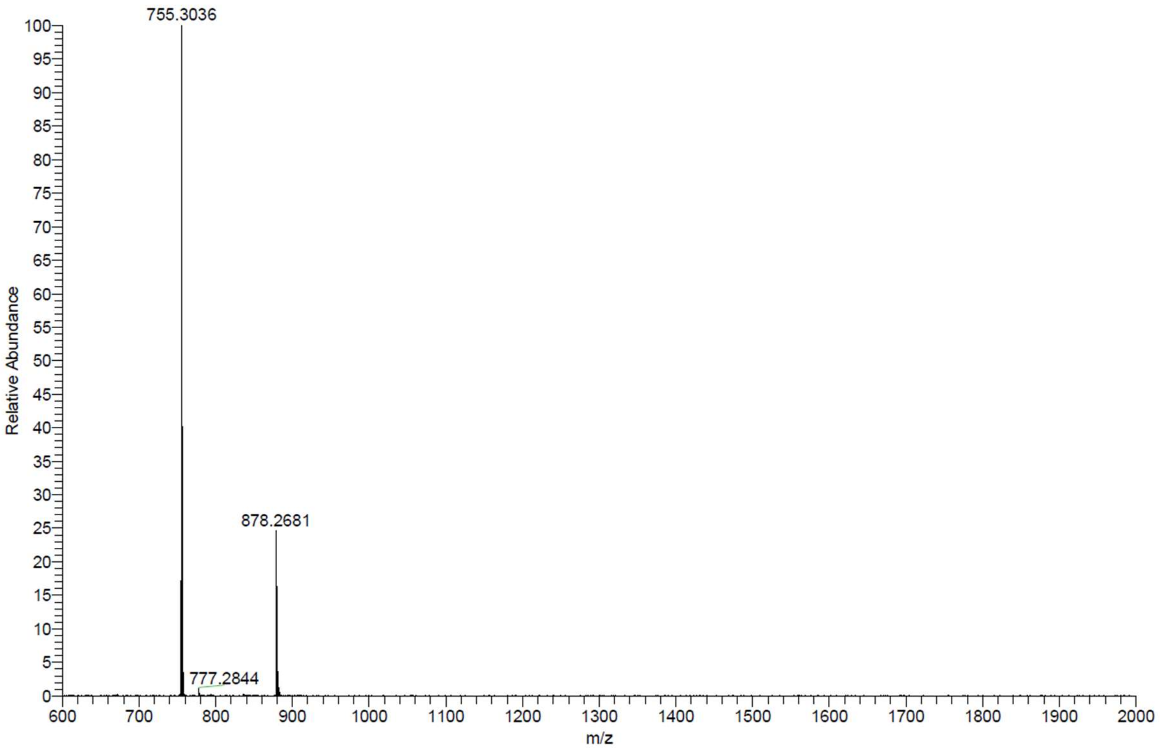

Figure S12 – HRMS spectrum fragment (APCI+) of **7**

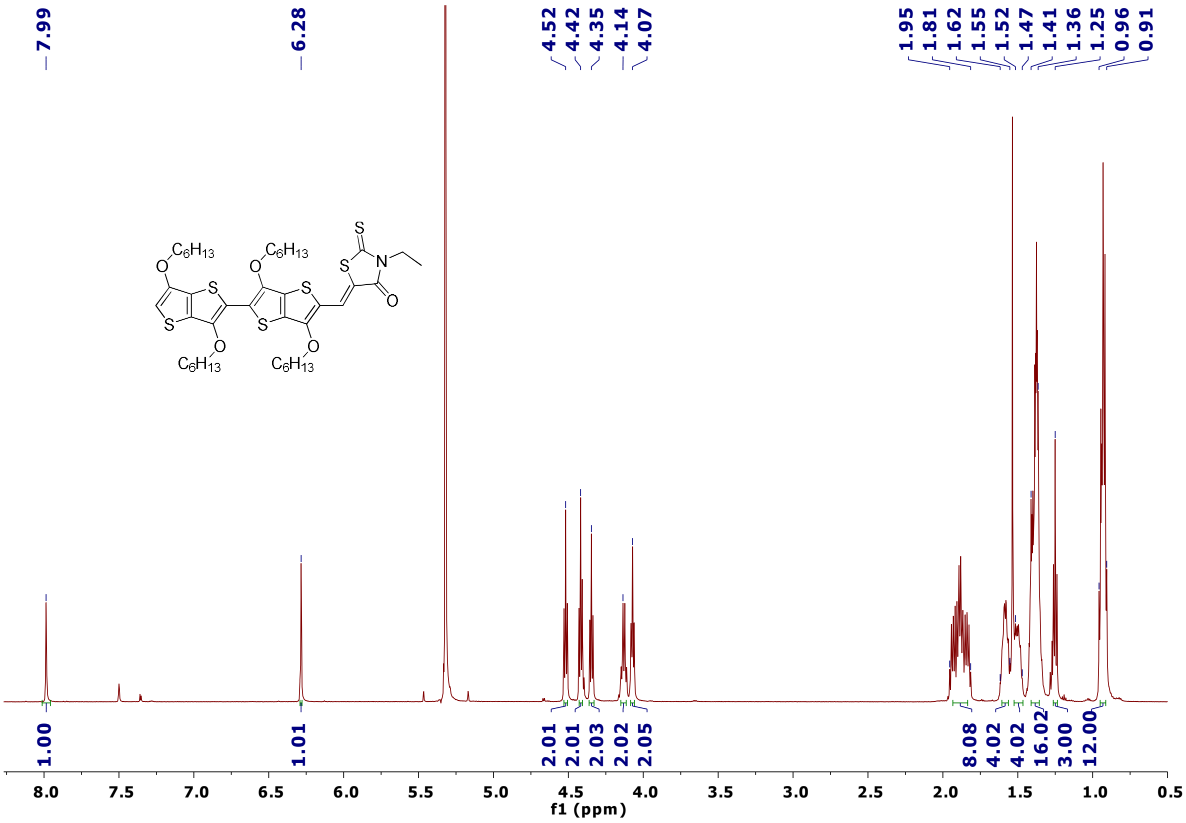

Figure S13 – ^{1}H-NMR spectrum fragment (600 MHz, CD_2Cl_2) of **8**

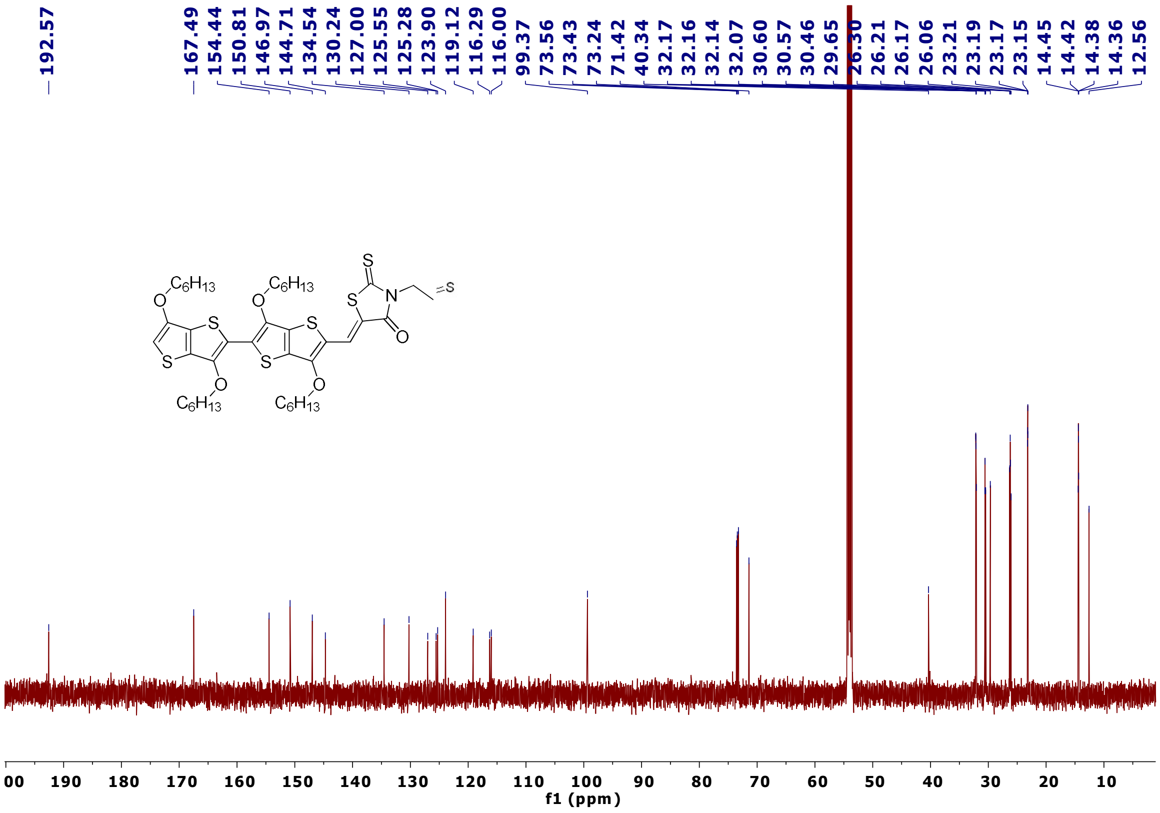

Figure S14 – ^{13}C-NMR spectrum fragment (150 MHz, CD_2Cl_2) of **8**

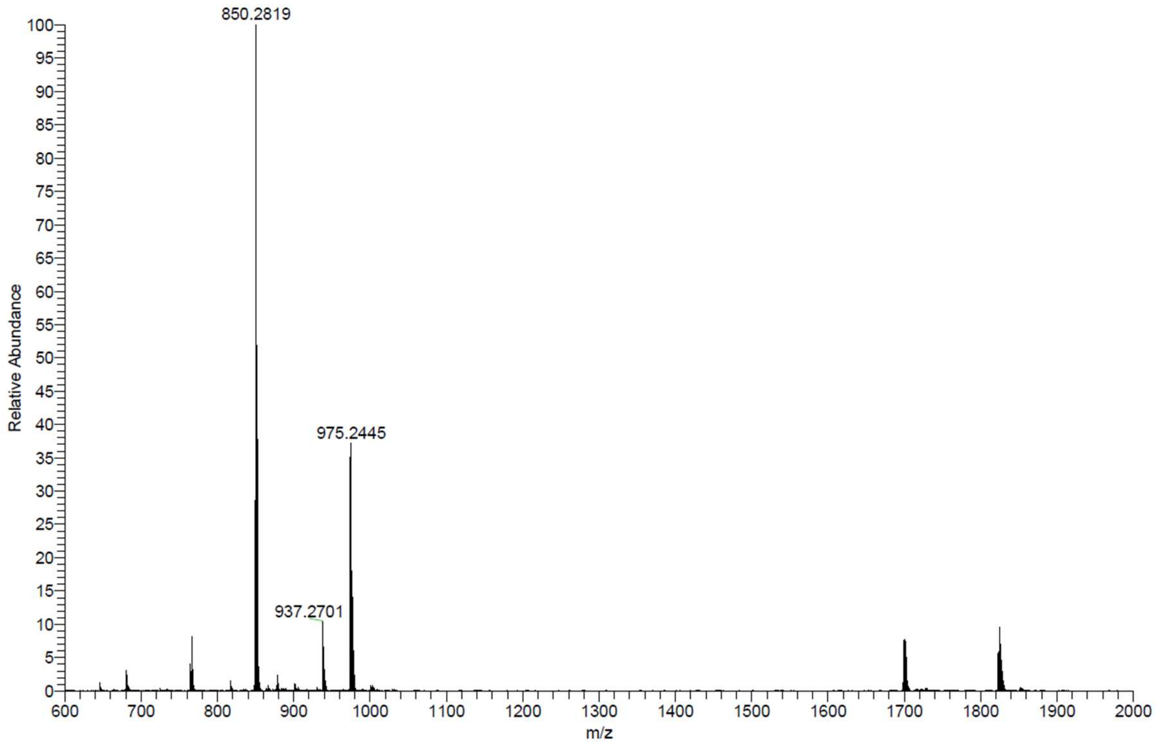

Figure S15 – HRMS spectrum fragment (APCI+) of **8**

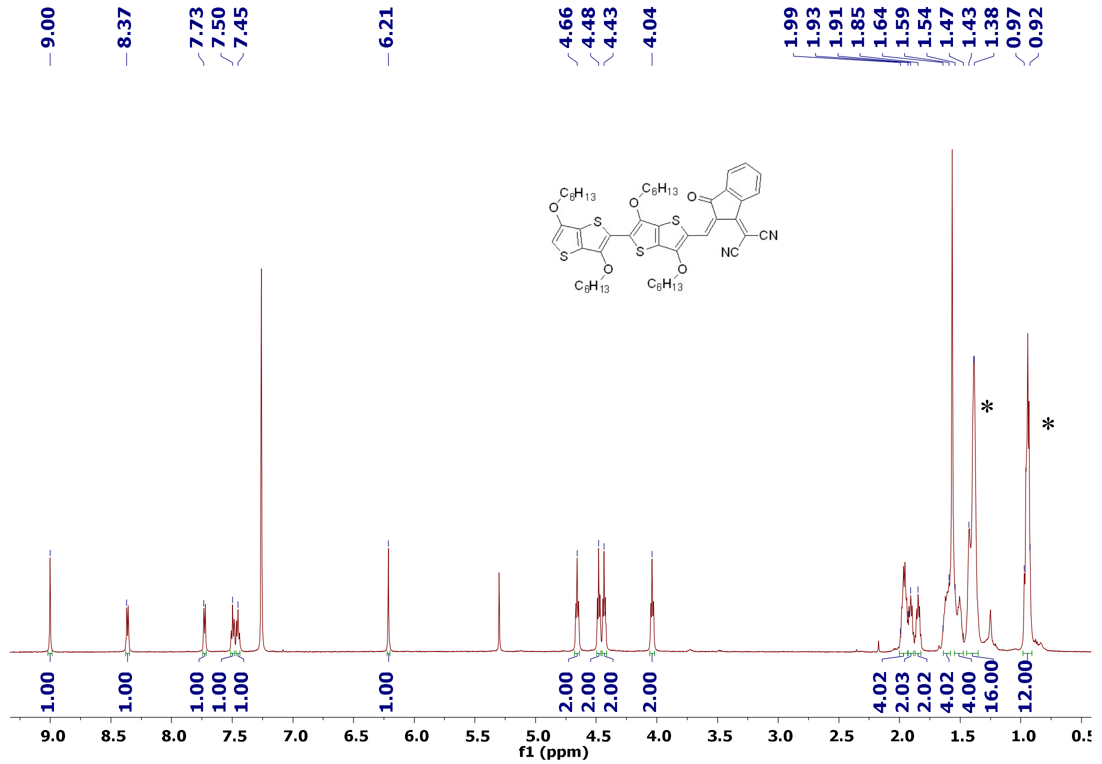

Figure S16 – ^{1}H-NMR spectrum fragment (600 MHz, $CDCl_3$) of **9** (*solvent residue)

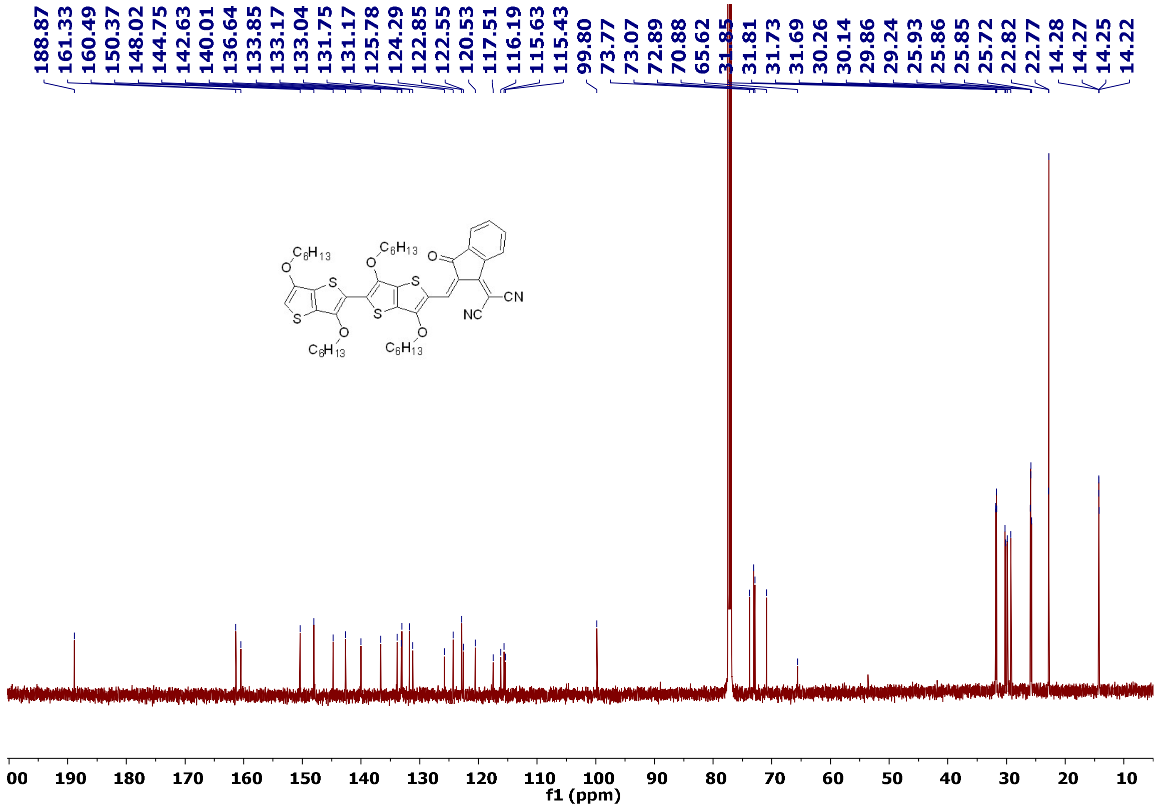

Figure S17 – ^{13}C-NMR spectrum fragment (150 MHz, $CDCl_3$) of **9**

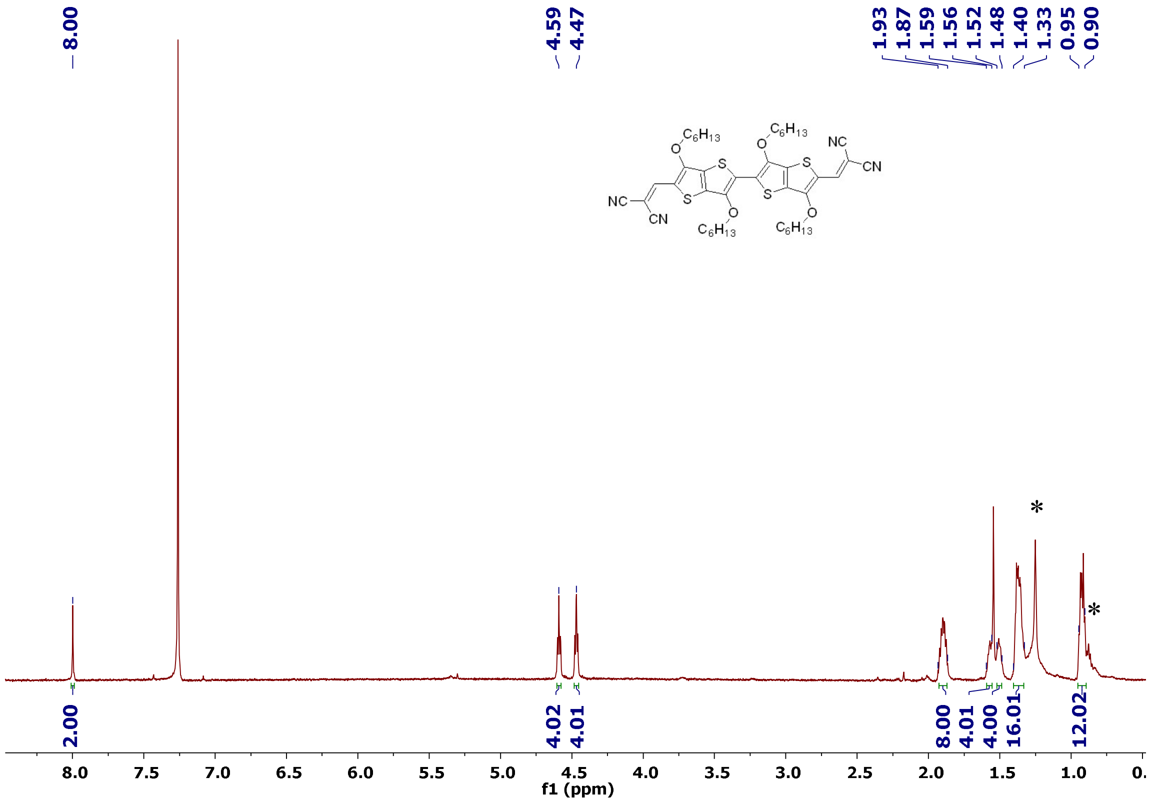

Figure S18 – ^{1}H-NMR spectrum fragment (600 MHz, $CDCl_3$) of **10** (*solvent residue)

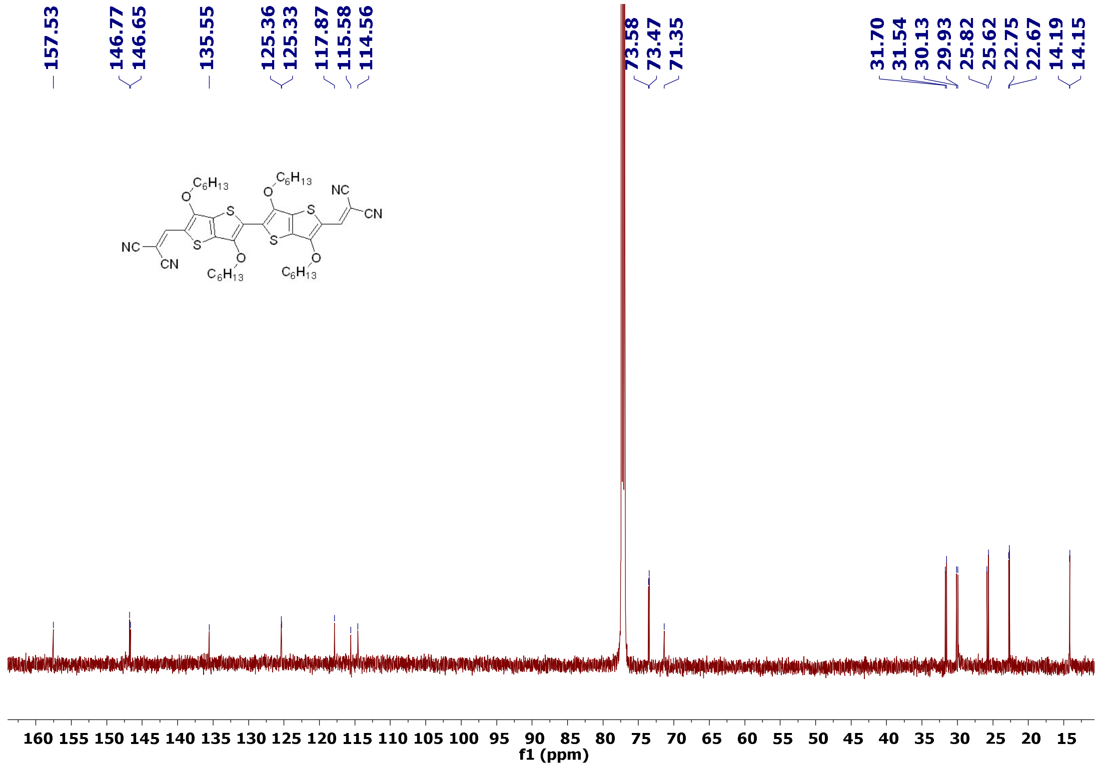

Figure S19 – ^{13}C-NMR spectrum fragment (150 MHz, $CDCl_3$) of **10**

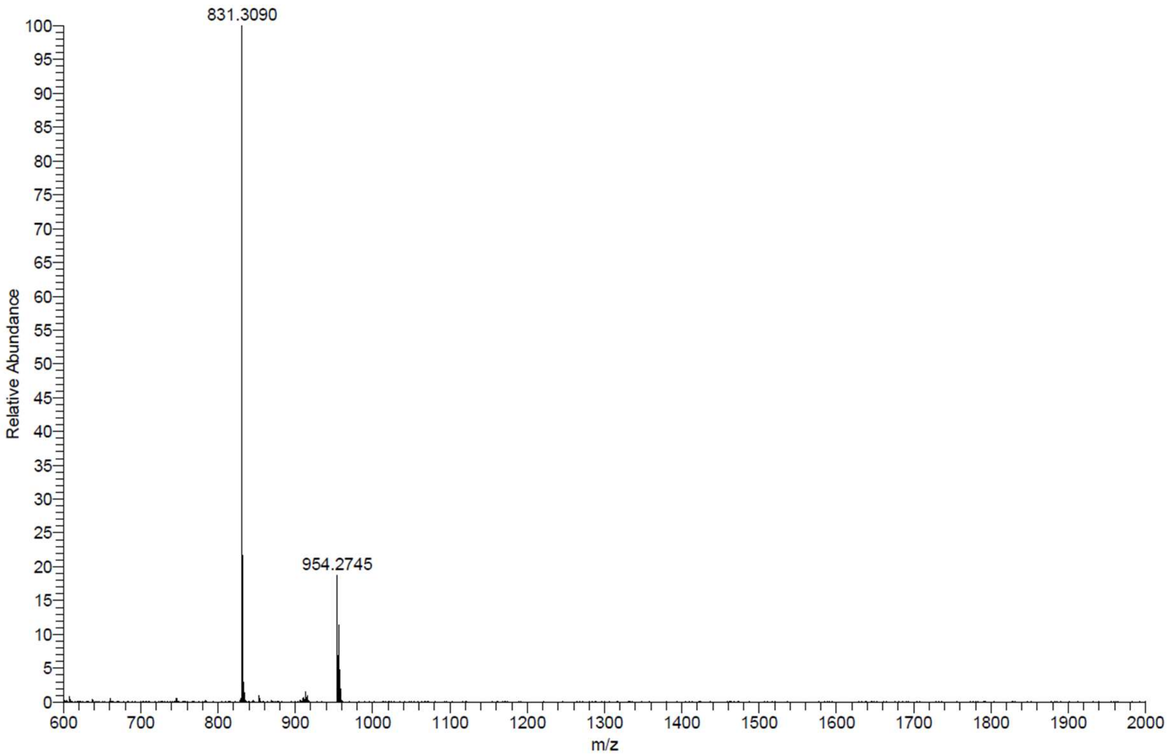

Figure S20 – HRMS spectrum fragment (APCI+) of **10**

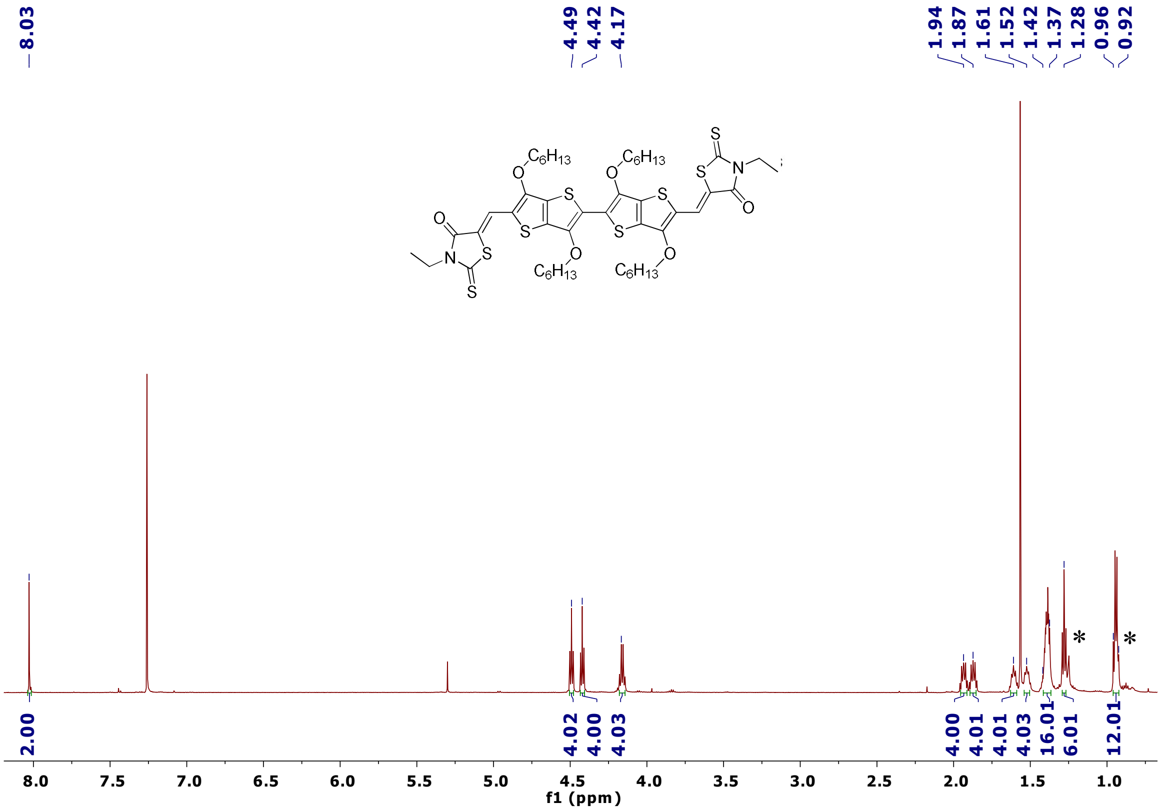

Figure S21 – ^{1}H-NMR spectrum fragment (600 MHz, $CDCl_3$) of **11** (*solvent residue)

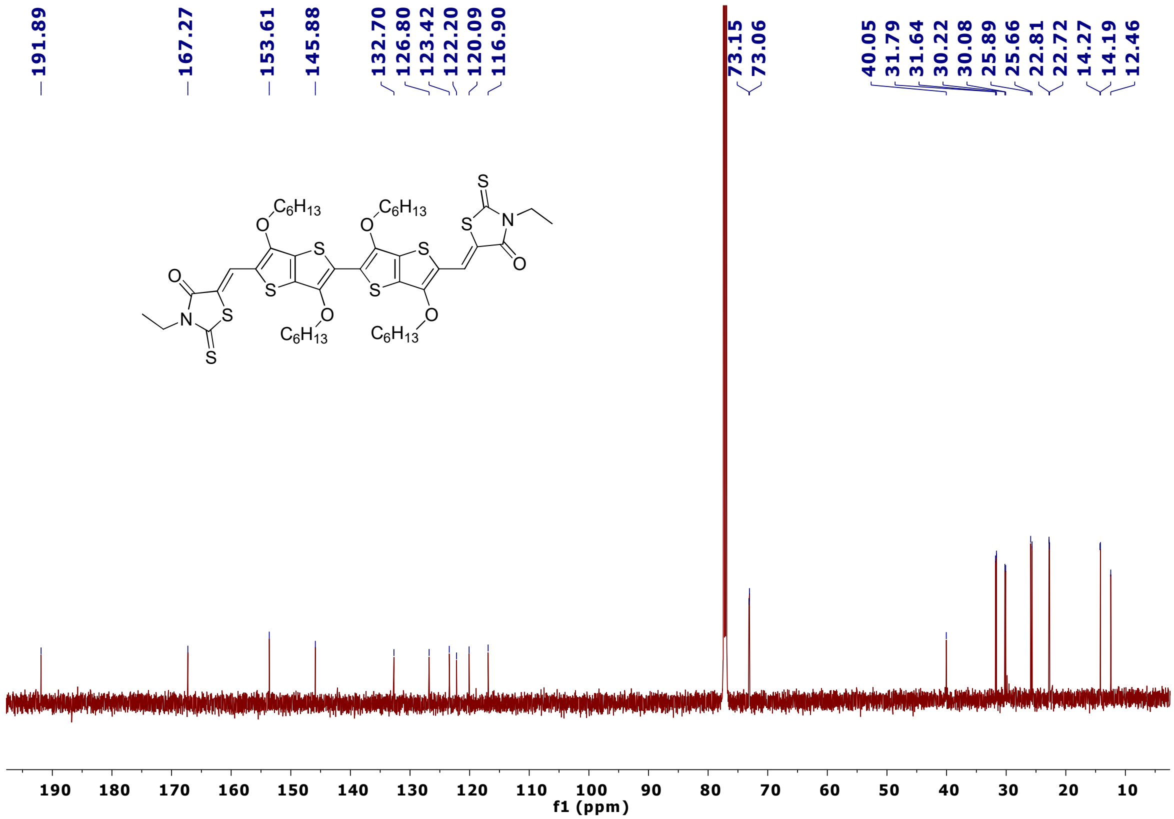

Figure S22 – ^{13}C-NMR spectrum fragment (150 MHz, $CDCl_3$) of **11**

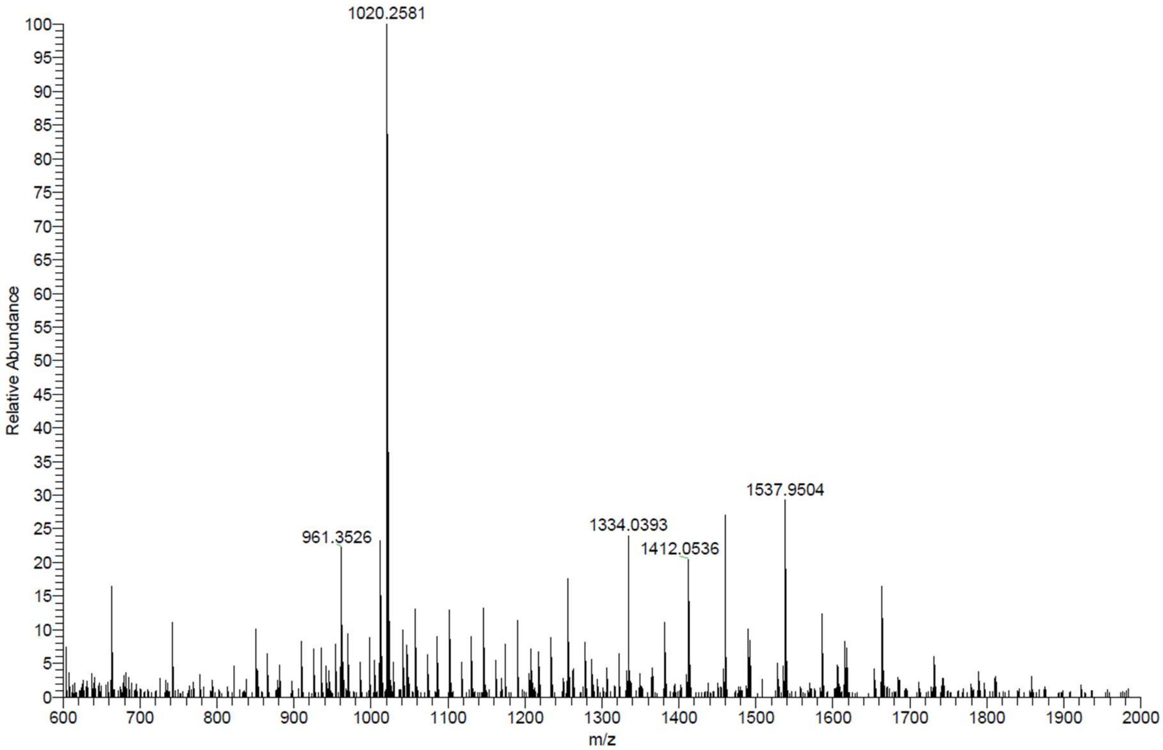

Figure S23 – HRMS spectrum fragment (APCI+) of 11

Computational details

Density functional theory (DFT) studies were performed using the Gaussian 09 package. Firstly, all geometries were fully optimized in gas phase without any symmetry constraints. As DFT method was chosen the B3LYP hybrid functional including with dispersion corrections (with D3 standing for Grimme's dispersion corrections) along with Def2-TZVP basis set. Analytic second derivative calculations were performed to characterize the nature of the stationary points. According to the vibrational analysis, all optimized geometries correspond to minima. Also, the optimization criteria were set to tight in all cases and the ultrafine integration grid was used for all calculations.

TD-DFT calculations were also performed in order to calculate the UV-vis absorbtion spectra. The first 30 electronic excited states were computed considering the B3LYP-D3 exchange-corelation functional and Def2-TZVP basis set. For a better understanding of occured electronic transition, natural transition orbitals (NTOs) were computed at the same level of theory for the most relevant excited states.

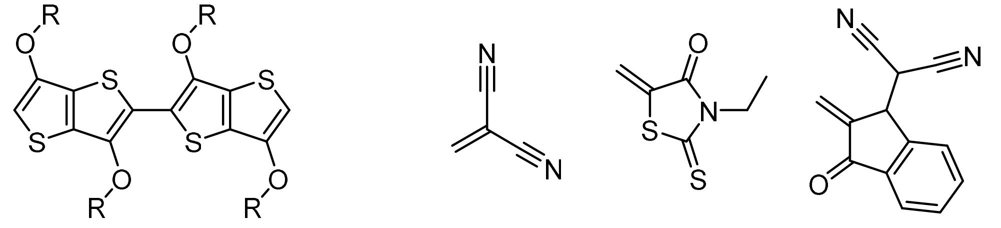

[3,2-*b*]-TT **EWG=** **(1)** **(2)** **(3)**

Table S1. HOMO-LUMO energy levels for the [3,2-*b*]-TT core and electron-withdrawing groups

Name	E_{HOMO} (eV)	E_{LUMO} (eV)
**[3,2-*b*]-TT**	-5.32	-1.77
**EWG=(1)**	-8.61	-2.89
**EWG=(2)**	-6.68	-2.70
**EWG=(3)**	-7.44	-3.53

Table S2. DFT coordinates for compounds 7-11

Compound **7**

E (298 K, 1 atm, solvent=$CHCl_3$) = -2774.220899 a.u.

E_{HOMO} = -0.20282 a.u.

E_{LUMO} = -0.10705 a.u.

S	5.31817900	-4.19278200	-1.28726500
C	4.17969700	-5.46470300	-1.00695300
C	3.04133100	-5.34477000	-1.75237500
C	3.08679800	-4.19223600	-2.59420800
C	4.26416300	-3.46544000	-2.46372600

C	4.27909500	-2.29991400	-3.27584900
C	3.11229600	-2.15159500	-4.01760500
S	1.98047500	-3.46751300	-3.70085200
C	2.80875100	-1.09678000	-4.93692200
C	1.62798900	-0.93732900	-5.65458300
C	1.64599900	0.19362000	-6.48972200
C	2.83407700	0.92354800	-6.41988300
S	3.95376600	0.18599100	-5.30536800
C	2.82705000	2.04287200	-7.27334800
C	1.61662800	2.15845200	-7.99731100
S	0.50235100	0.84578800	-7.59985400
O	5.24219700	-1.36426400	-3.37261700
C	6.56107100	-1.65168300	-2.89365000
O	1.99183400	-6.19917600	-1.62192000
C	1.56463700	-6.86227700	-2.82143100
O	0.60356900	-1.83047900	-5.58218600
C	-0.57852800	-1.36253300	-4.90192400
O	3.78062700	2.95511800	-7.46858500
C	5.00537100	2.87979100	-6.72610300
H	6.57505300	-1.69912700	-1.80347100
H	7.18177200	-0.82345400	-3.22421000
H	6.93430200	-2.58376600	-3.32035800
H	0.78508200	-7.55753100	-2.51988000
H	2.39540900	-7.41014200	-3.27267200
H	1.15601600	-6.15729600	-3.54880200
H	-1.28358700	-2.18939100	-4.92003200
H	-1.00925100	-0.50076600	-5.41495300
H	-0.34109200	-1.09802600	-3.86954500
H	5.59647100	3.73118500	-7.05012600
H	5.53876800	1.95654100	-6.95647300
H	4.81188900	2.95546700	-5.65519000
C	1.37906400	3.19680700	-8.90179100
H	2.20356100	3.89418600	-8.99624400
C	0.27788100	3.47887700	-9.68697500
C	-0.90711200	2.69938900	-9.71304000
N	-1.87573000	2.06983800	-9.73808800
C	0.29569800	4.61806700	-10.53548100
N	0.31130500	5.54503400	-11.22506700
H	4.39747300	-6.24596900	-0.29688100

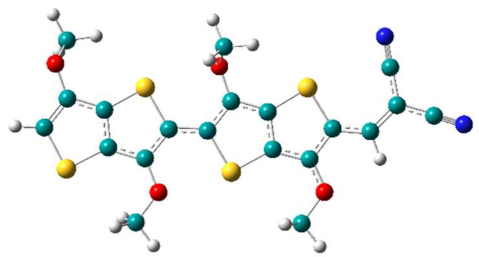

Compound **8**
E (298 K, 1 atm, solvent=DCM) = -3670.476156 a.u.
E_{HOMO} = -0.19761 a.u.
E_{LUMO} = -0.10515 a.u.

S	5.64857900	-3.95714800	-1.26578300
C	4.58744300	-5.31609300	-0.89928200

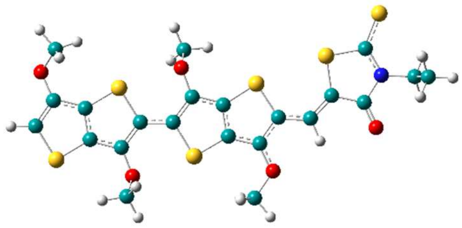

C	3.40338200	-5.25910800	-1.65480600
C	3.37016500	-4.13668700	-2.51481800
C	4.51703000	-3.35256800	-2.41411000
C	4.50513000	-2.21765300	-3.25186800
C	3.34254900	-2.11725800	-3.99903100
S	2.25098700	-3.45498400	-3.66394800
C	3.00416500	-1.07933700	-4.93252100
C	1.84583900	-0.98431200	-5.68264600
C	1.82081100	0.16762700	-6.50815400
C	2.95845600	0.95340100	-6.38788400
S	4.08336000	0.27726000	-5.25667900
C	2.94057800	2.08674600	-7.26069600
C	1.79554600	2.12316900	-8.00851900
S	0.73135600	0.79099000	-7.69777100
O	5.48832800	-1.27754500	-3.29446300
C	6.71197800	-1.69143500	-3.93565700
O	2.48242100	-6.21535900	-1.48592200
C	1.29351700	-6.20670600	-2.28652600
O	0.89066600	-1.94931800	-5.64574400
C	-0.41199600	-1.53540300	-5.19114700
O	3.92260700	3.00874400	-7.42973400
C	4.59349700	3.47208500	-6.25104800
H	7.16080900	-2.53747400	-3.41205900
H	7.38037800	-0.83569400	-3.89024700
H	6.52101700	-1.96018100	-4.97646000
H	0.73086100	-7.08558100	-1.98555000
H	1.54049200	-6.27785900	-3.34685100
H	0.70127800	-5.31169400	-2.08969700
H	-1.02323400	-2.43382900	-5.17480900
H	-0.85329400	-0.80455900	-5.87062700
H	-0.34454900	-1.11150700	-4.18731800
H	5.17768100	4.33639500	-6.55738900
H	5.26855600	2.71198700	-5.85108000
H	3.87437100	3.76678200	-5.48378300
C	4.87158200	-6.33824700	0.02680600
H	4.09804000	-7.09388900	0.11990500
C	5.95220200	-6.54605200	0.82853400
S	7.40954100	-5.58074200	0.99568900
C	6.01736000	-7.70289200	1.71989000
C	8.08459200	-6.66613100	2.19381800
N	7.23372400	-7.70190200	2.43146900
O	5.17479800	-8.57127400	1.86614000
S	9.56051700	-6.39593800	2.88966500
C	7.51013400	-8.76241000	3.40715500
H	8.58267400	-8.94366200	3.39305100
H	6.99599400	-9.65066000	3.04633900
C	7.03322700	-8.38593500	4.80402500

H	5.95724400	-8.20887700	4.80919900
H	7.54097900	-7.48749600	5.15535500
H	7.25171000	-9.19914400	5.49757700
H	1.53408000	2.87524700	-8.73486500

Compound **9**
E (298 K, 1 atm, solvent=$CHCl_3$) = -3194.984835 a.u.
E_{HOMO} = -0.2007 a.u.
E_{LUMO} = -0.11695 a.u.

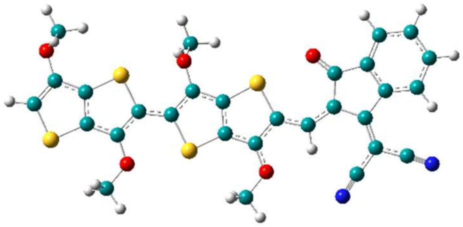

S	7.42309200	-6.39828600	-6.81515900
C	6.45337100	-7.77492500	-6.26229600
C	5.05810300	-7.48052300	-6.35522700
C	4.80712000	-6.19804200	-6.85994300
C	5.98643900	-5.50784900	-7.15452000
C	5.76300800	-4.21294900	-7.65220400
C	4.41189100	-3.88759300	-7.73745500
S	3.40103100	-5.21948100	-7.19390600
C	3.86252200	-2.64598000	-8.18781100
C	2.51379800	-2.32207000	-8.29672300
C	2.30742500	-0.99875500	-8.76818300
C	3.49884600	-0.32586500	-9.01578400
S	4.88598400	-1.29642100	-8.68235800
C	3.30124600	0.99194200	-9.53037800
C	1.97248100	1.28419300	-9.65861800
S	0.93562700	-0.01594500	-9.18428800
O	6.74266800	-3.32195200	-7.96906900
C	7.49823600	-3.62770700	-9.15771500
O	4.18758300	-8.40970800	-5.96796700
C	2.77852800	-8.17203000	-6.07564800
O	1.58798600	-3.24987100	-7.99196400
C	0.23864700	-2.84582100	-7.72971300
O	4.26923500	1.85839300	-9.92783900
C	5.30802700	2.13819400	-8.97849800
H	8.02700400	-4.57654500	-9.05158900
H	8.21567800	-2.81967000	-9.27475500
H	6.83776500	-3.66755400	-10.02639100
H	2.30317700	-9.08113800	-5.72016600
H	2.49743500	-7.99213300	-7.11431200
H	2.48141200	-7.33240800	-5.44557500
H	-0.26238300	-3.72747000	-7.33963800
H	-0.25604300	-2.52824300	-8.64908500
H	0.21051800	-2.04746000	-6.98682500
H	5.89833200	2.94387400	-9.40802000
H	5.95149300	1.27045300	-8.81733200
H	4.88244700	2.45934000	-8.02490200
C	6.86674800	-9.01779100	-5.78479700

C	8.10127300	-9.61332500	-5.57218100
C	9.41063000	-8.99683200	-5.82916300
C	8.32961200	-10.95052700	-5.06128300
C	10.43525000	-9.99759500	-5.46465300
O	9.63563600	-7.87527800	-6.26020700
C	9.80054900	-11.16017200	-5.00876400
C	7.39708400	-11.89956700	-4.67622200
C	11.80976000	-9.87828400	-5.53503000
C	10.58061200	-12.24257900	-4.60938000
C	5.98706100	-11.71699100	-4.71349200
C	7.73736800	-13.19079500	-4.18772200
C	12.58416300	-10.96434900	-5.13424600
H	12.25794900	-8.96085900	-5.89346900
C	11.96928300	-12.12872300	-4.67829400
H	10.14832300	-13.16153700	-4.25045700
N	4.83485100	-11.63187600	-4.71825000
N	7.94887800	-14.25258800	-3.78506200
H	13.66403200	-10.90717900	-5.17569200
H	12.57912100	-12.96767800	-4.36902300
H	6.00921300	-9.62626600	-5.53621900
H	1.55661300	2.21088300	-10.01949900

Compound **10**
E (298 K, 1 atm, solvent=$CHCl_3$) = -3036.222532 a.u.
E_{HOMO} = -0.21384 a.u.
E_{LUMO} = -0.126 a.u.

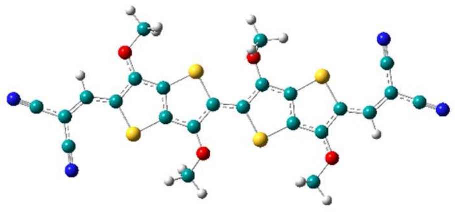

S	5.41301100	-4.10040600	-1.23045100
C	4.36526500	-5.49470400	-0.98628200
C	3.21417500	-5.42729000	-1.79757500
C	3.19762800	-4.26655300	-2.60217900
C	4.32279200	-3.46225900	-2.40681400
C	4.30525300	-2.28773600	-3.19337000
C	3.16583300	-2.19682300	-3.98581300
S	2.09940000	-3.57131800	-3.75482000
C	2.85014500	-1.13895600	-4.90058800
C	1.69593100	-1.03434800	-5.66651400
C	1.69163700	0.11235200	-6.48598200
C	2.83782600	0.89562900	-6.34770400
S	3.94300400	0.20470100	-5.19451800
C	2.81865000	2.02827400	-7.19073800
C	1.64292400	2.09154700	-7.96925200
S	0.57571900	0.72486700	-7.64504100
O	5.20863800	-1.28771900	-3.20355500
C	6.60087600	-1.60669400	-3.03742500
O	2.31308600	-6.40592300	-1.72042400
C	1.18440200	-6.42224600	-2.60743100

O	0.73459000	-1.99074400	-5.64454700
C	-0.55594400	-1.58496500	-5.14177500
O	3.73495800	2.98611500	-7.32776700
C	4.92875200	2.95914600	-6.53160800
H	6.82968000	-1.81285700	-1.99097200
H	7.14578300	-0.72179400	-3.35468200
H	6.87805500	-2.45732600	-3.66100700
H	0.64967500	-7.33959000	-2.38049900
H	1.51319200	-6.43732000	-3.64715900
H	0.53468800	-5.56663100	-2.41903500
H	-1.17287700	-2.47922800	-5.14927000
H	-1.00554000	-0.82447400	-5.78166300
H	-0.46062500	-1.20360700	-4.12340800
H	5.49038200	3.84300700	-6.81848200
H	5.51624700	2.06658000	-6.75070900
H	4.68408400	3.01045000	-5.46992000
C	1.39898600	3.13309800	-8.87583600
H	2.19147300	3.87121200	-8.92012000
C	0.32750000	3.36774100	-9.70884000
C	4.60587200	-6.56490400	-0.11093900
H	3.83111900	-7.32279000	-0.12054400
C	5.65495100	-6.80375900	0.74774600
C	-0.81264900	2.52657000	-9.80237700
N	-1.74271400	1.84648500	-9.88145200
C	0.32550300	4.51877900	-10.54376600
N	0.32402800	5.45369100	-11.22155100
C	5.66155000	-7.98609200	1.53814600
N	5.66701800	-8.94592400	2.17989700
C	6.76711700	-5.93621500	0.91323300
N	7.67339600	-5.23391100	1.05111900

Compound **11**
E (298 K, 1 atm, solvent=$CHCl_3$) = -4828.730855 a.u.
E_{HOMO} = -0.19934 a.u.
E_{LUMO} = -0.1161 a.u.

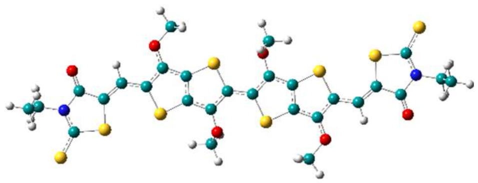

S	5.60368500	-3.89956600	-1.23229200
C	4.57305500	-5.29007700	-0.91033000
C	3.40378300	-5.25328500	-1.68526200
C	3.35704900	-4.11726300	-2.52946500
C	4.48062100	-3.30436600	-2.39392700
C	4.44846400	-2.15529600	-3.21288800
C	3.29589300	-2.07878700	-3.97928300
S	2.23797800	-3.45256500	-3.68564600
C	2.94942400	-1.03651300	-4.90243700
C	1.80114400	-0.96466800	-5.67576900
C	1.76603100	0.18795200	-6.48974100

C	2.88129500	1.00969700	-6.34031700
S	3.99986300	0.34569500	-5.18368900
C	2.83363800	2.14839200	-7.18067900
C	1.67263100	2.17796800	-7.96793500
S	0.65221800	0.77582900	-7.66418600
O	5.38988800	-1.17691300	-3.23039400
C	6.68375800	-1.55371200	-3.74314400
O	2.50556900	-6.23713300	-1.54938200
C	1.32461300	-6.23998800	-2.36003300
O	0.86694500	-1.94993100	-5.66782500
C	-0.43616600	-1.58240700	-5.17236400
O	3.72832900	3.13697300	-7.30666400
C	4.84638700	3.20154500	-6.41315400
H	7.14876100	-2.31611500	-3.11613400
H	7.28667900	-0.64983900	-3.72637500
H	6.59170800	-1.92285700	-4.76641700
H	0.78062800	-7.13819000	-2.08248400
H	1.58136300	-6.28348200	-3.41967100
H	0.70868000	-5.36382900	-2.15045200
H	-1.03129300	-2.49133000	-5.19397100
H	-0.89985300	-0.82567400	-5.80713500
H	-0.35985500	-1.20968400	-4.14909800
H	5.37131200	4.11824200	-6.66591200
H	5.51282700	2.35046800	-6.56355500
H	4.51154600	3.24705400	-5.37565500
C	1.37386600	3.21447100	-8.87871700
H	2.11711500	4.00375100	-8.92845500
C	0.31073100	3.39476200	-9.70587600
S	-1.09879900	2.37654800	-9.94183700
C	-1.77875900	3.46142900	-11.14220800
C	0.22267300	4.57571500	-10.57052700
N	-0.95487000	4.52861900	-11.33996900
O	1.02229100	5.48957400	-10.64928600
S	-3.22263800	3.15493400	-11.88013300
C	-1.26736000	5.62440900	-12.26480800
H	-1.79713000	5.19213800	-13.11095000
H	-0.31206000	6.01714600	-12.60664300
C	-2.09555500	6.71238800	-11.59354200
H	-1.55587300	7.14052500	-10.74822900
H	-3.04560400	6.31054900	-11.24090500
H	-2.30281000	7.50956800	-12.30881400
C	4.86945700	-6.32236600	0.00583200
H	4.11345800	-7.09777000	0.07692000
C	5.94432900	-6.51419300	0.81516400
S	7.37703400	-5.52062800	1.01408300
C	6.02604100	-7.68734500	1.69081700
C	8.06555400	-6.61345100	2.20222100

N	7.23391000	-7.67206800	2.41319200
O	5.20001100	-8.57273000	1.81165400
S	9.52527800	-6.32316000	2.91528400
C	7.52397900	-8.74304900	3.37368100
H	8.60051300	-8.89962400	3.36772200
H	7.03279900	-9.63579400	2.99229400
C	7.02465100	-8.40098900	4.77151900
H	5.94492800	-8.24846300	4.76816500
H	7.50906600	-7.49792600	5.14329100
H	7.25410800	-9.22083200	5.45365800

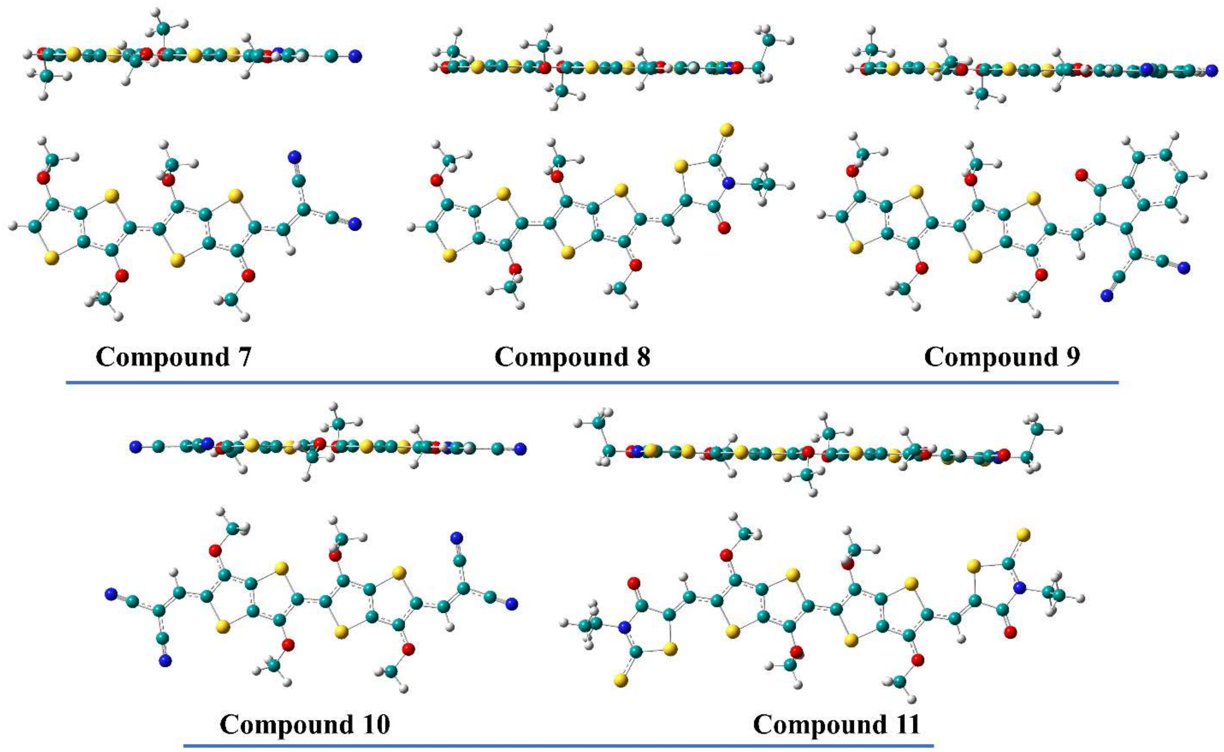

Figure S24 - Side view and top view of the optimized geometries of compounds **7-11**.
